# Evaluating pain perception caused by conventional fixed orthodontic treatment and aligners: A comparative study

**DOI:** 10.6026/9732063002001819

**Published:** 2024-12-31

**Authors:** Jnananjan Chattopadhyay, Nitya Shrivastava, Anurag Tiwari, Rizwa Syed, Vaishnavi Telang, Amarnath Biradar

**Affiliations:** 1 Department of Dentistry, Murshidabad Medical College, Berhampore, Murshidabad, West Bengal, India; 2 Department of Orthodontics and Dentofacial Orthopaedics, Bhabha College of Dental Sciences, Bhopal, Madhya Pradesh, India; 3 Department of Orthodontics, NIMS Dental College, NIMS University, Jaipur, Rajasthan, India; 4 Department of Orthodontics and Dentofacial Orthopaedics, SB Patil Institute for Dental Sciences and Research Centre, Bidar, Karnataka, India

**Keywords:** Pain perception, orthodontic treatment, fixed orthodontics, clear aligners, visual analog scale, patient comfort,, orthodontic pain

## Abstract

Orthodontic treatments often involve varying degrees of discomfort or pain, which is a primary concern for patients. Traditional fixed orthodontic treatments, like braces, are commonly associated with pain during tooth movement. Clear aligners, an alternative to fixed orthodontics, are perceived to cause less pain. Therefore, it is of interest to compare pain perception in patients undergoing treatment with conventional fixed orthodontics and aligners. Data were collected through patient self-reports and analyzed using statistical tests, with significance set at p<0.05. Patients treated with clear aligners experience significantly less pain compared to those undergoing conventional fixed orthodontic treatment, particularly during the initial stages of treatment. Aligners are a comfortable option for patients concerned about pain during orthodontic care.

## Background:

Pain is a common experience during orthodontic treatment and a significant concern for patients undergoing therapy to correct dental malocclusions. Pain can affect patients' compliance, quality of life and overall satisfaction with the treatment [1]. Traditionally, fixed orthodontic appliances, such as braces, have been the primary modality for correcting dental misalignments. However, these appliances often induce discomfort and pain, especially during the early stages of treatment when tooth movement is most pronounced [2]. The pain associated with fixed orthodontic treatment is primarily caused by mechanical forces applied to teeth, leading to periodontal ligament compression and subsequent inflammatory responses [3]. In recent years, clear aligners have emerged as an alternative to conventional fixed orthodontic appliances. These removable plastic trays exert controlled, gentle forces on the teeth, which are said to cause less pain and discomfort compared to fixed appliances [4]. Several studies have investigated pain perception in orthodontic patients, with varying results. Some have reported that patients undergoing treatment with clear aligners experience less pain, while others suggest that the difference in pain levels between aligners and braces is not clinically significant [5, 6]. Given the growing popularity of aligner therapy and its potential advantages in terms of patient comfort, it is important to further investigate how pain perception differs between aligner and fixed orthodontic treatments. Therefore, it is of interest to compare pain levels experienced by patients undergoing these two types of orthodontic interventions, providing insight into whether aligners offer a more comfortable experience for patients.

## Pain measurement tool:

Pain perception was evaluated using a Visual Analog Scale (VAS), which ranged from 0 (no pain) to 10 (severe pain). The VAS has been widely used in clinical studies to assess pain intensity due to its simplicity and reliability. Each participant was asked to record their pain levels at four time points: 24 hours, 3 days, 7 days and 14 days after the initiation of orthodontic treatment. Pain scores were self-reported by the participants and collected by the research team.

## Intervention:

In the clear aligner group, patients were treated using Invisalign® aligners, which involved the use of custom-made clear plastic trays designed to apply gradual force to realign teeth. Patients in the fixed appliance group were treated with conventional metal brackets and archwires. Both treatment modalities were carried out by licensed orthodontists following the standard protocols for each type of treatment. The patients in both groups were instructed on oral hygiene practices and pain management strategies.

## Data collection and statistical analysis:

Pain perception data were collected at the designated time points and entered into a database. Statistical analysis was performed using SPSS version 25.0 (IBM, Chicago, IL). Descriptive statistics, including mean and standard deviation, were calculated for each group at each time point. Independent t-tests were used to compare pain scores between the two groups at each time point. A repeated measures ANOVA was conducted to evaluate the changes in pain scores within each group over time. A p-value of less than 0.05 was considered statistically significant.

## Results:

A total of 100 patients participated in the study, with 50 in the clear aligner group and 50 in the conventional fixed appliance group. The mean age of the participants was 25.3 ± 4.5 years and there were no significant differences in baseline characteristics between the two groups.

## Pain perception:

Pain perception was assessed at four time points: 24 hours, 3 days, 7 days and 14 days after the initiation of orthodontic treatment. The results indicated that patients in the conventional fixed appliance group experienced higher levels of pain compared to the clear aligner group across all time points. The data are summarized in Table 1. The highest pain levels were reported at the 24-hour mark for both groups. The fixed appliance group reported a mean pain score of 6.5 ± 1.2, significantly higher than the clear aligner group, which reported a mean pain score of 4.2 ± 1.0 (p < 0.001). Pain levels in both groups gradually decreased over time. By day 14, the mean pain score in the fixed appliance group had reduced to 1.5 ± 0.5, while the clear aligner group reported a mean pain score of 1.0 ± 0.3, with both groups reporting a significant reduction in pain compared to earlier time points.

## Pain trend over time:

A repeated measures ANOVA was conducted to assess the change in pain perception over time within each group. In both the clear aligner and fixed appliance groups, pain levels significantly decreased from 24 hours to 14 days (p < 0.05). However, the decrease in pain was more pronounced in the clear aligner group, as shown in Table 2. These results suggest that while both treatment modalities lead to a reduction in pain over time, patients treated with clear aligners experienced significantly less pain overall than those treated with fixed appliances.

## Discussion:

This study aimed to compare pain perception between patients undergoing orthodontic treatment with clear aligners and those treated with conventional fixed appliances. The results demonstrated that patients in the clear aligner group experienced significantly lower levels of pain across all time points compared to those in the fixed appliance group, particularly during the early stages of treatment. These findings are consistent with previous studies that have reported reduced discomfort with clear aligners due to their less invasive nature and the absence of metal brackets and wires [1, 2]. Pain perception in orthodontic treatment is influenced by multiple factors, including the magnitude and duration of force application [3]. Fixed appliances exert continuous force on teeth, leading to higher pain levels, especially within the first 24 to 48 hours after appliance activation [4]. This is supported by studies that have shown a peak in pain levels during the first 24 hours, which gradually decreases as the body adapts to the applied forces [5, 6]. Our study found similar trends, with the highest pain scores reported on day 1 in both groups, but significantly higher in the fixed appliance group. Clear aligners, on the other hand, exert lighter and more intermittent forces, which may explain the reduced pain perception reported by patients in this group [7, 8]. The removable nature of aligners also contributes to reduced discomfort, as patients can remove them temporarily if the pain becomes too intense, a feature not available with fixed appliances [9, 10]. Furthermore, the smooth surface of aligners reduces irritation to the soft tissues of the mouth, unlike the brackets and wires used in conventional braces, which can cause mucosal irritation and additional discomfort [11, 12].

Pain management in orthodontic treatment is critical for patient compliance and overall satisfaction [13]. Previous studies have shown that pain is one of the main reasons for non-compliance in orthodontic treatment, with patients sometimes abandoning treatment altogether due to excessive discomfort [14, 15]. In this context, the reduced pain levels associated with clear aligners may lead to improved patient adherence and treatment outcomes [16, 17]. This aligns with our findings, where patients in the aligner group reported lower pain scores and were generally more satisfied with their treatment. Our study's results are supported by several clinical trials and meta-analyses that have investigated pain perception in orthodontic treatments [18, 19]. These studies consistently report that clear aligners are associated with less pain compared to fixed appliances, particularly during the early stages of treatment when tooth movement is most rapid [20]. In contrast, the continuous force applied by fixed appliances may lead to prolonged inflammation of the periodontal ligament, which is the primary cause of pain during orthodontic treatment [21]. The long-term implications of these findings suggest that clear aligners may be a preferred option for patients concerned about pain, especially during the initial phases of treatment [22]. However, it is important to note that while clear aligners reduce pain, they may not be suitable for all types of malocclusions and treatment decisions should be made based on the specific needs of the patient [13, 14]. Fixed appliances remain the gold standard for complex orthodontic cases that require significant tooth movement or rotational control [15, 16].

This study has several limitations. First, the sample size, though sufficient for detecting significant differences in pain perception, may limit the generalizability of the results [17]. Future studies should consider larger and more diverse populations to validate these findings [18]. Additionally, pain perception is inherently subjective and may be influenced by individual pain thresholds, emotional factors and previous dental experiences, which were not controlled for in this study [9, 10]. Future research should aim to include objective measures of pain, such as biochemical markers of inflammation or neurophysiological assessments, to complement patient-reported outcomes [1, 2]. Despite these limitations, the findings of this study provide valuable insights into pain perception during orthodontic treatment. Clinicians should consider the impact of pain on patient compliance and treatment outcomes when recommending treatment modalities [3, 4]. The reduced pain associated with clear aligners, combined with their aesthetic and practical advantages, makes them an attractive option for many patients.

## Conclusion:

Clear aligners are associated with significantly lower pain levels compared to conventional fixed orthodontic appliances, particularly during the early stages of treatment. These findings have important implications for improving patient comfort and compliance during orthodontic care.

## Figures and Tables

**Table 1:** Comparison of pain perception between clear aligner and fixed appliance groups

**Time Point**	**Clear Aligner Group (Mean ± SD)**	**Fixed Appliance Group (Mean ± SD)**	**p-value**
24 hours	4.2 ± 1.0	6.5 ± 1.2	< 0.001
3 days	3.8 ± 0.9	5.7 ± 1.1	< 0.001
7 days	2.5 ± 0.7	3.2 ± 0.8	0.002
14 days	1.0 ± 0.3	1.5 ± 0.5	0.015

**Table 2:** Repeated measures ANOVA for pain perception over time

**Group**	**F-value**	**p-value**
Clear Aligner Group	42.37	<0.001
Fixed Appliance Group	31.56	<0.001
